# Defining activities in neurovascular microsurgery training: entrustable professional activities for vascular neurosurgery

**DOI:** 10.1007/s00701-022-05372-x

**Published:** 2022-10-22

**Authors:** Jasper Hans van Lieshout, Bastian Malzkorn, Hans-Jakob Steiger, Cihat Karadag, Marcel A. Kamp, Peter Vajkoczy, Jürgen Beck, Simone Peschillo, Veit Rohde, Daniel Walsh, Vasiliy Lukshin, Miikka Korja, Marco Cenzato, Andreas Raabe, Andreas Gruber, Daniel Hänggi, H. D. Boogaarts

**Affiliations:** 1 Department of Neurosurgery, Medical Faculty, Heinrich Heine University Düsseldorf, Moorenstraße 5, Düsseldorf, Germany; 2 Medical Education, Office of the Deanery of the Medical Faculty, Heinrich Heine University Düsseldorf, Düsseldorf, Germany; 3 Department of Neurosurgery, Medical Faculty, Friedrich Schiller University, Jena, Germany; 4 Department of Neurosurgery, Charité-Universitätsmedizin Berlin, Berlin, Germany; 5 Department of Neurosurgery, Medical Center and Faculty of Medicine, University of Freiburg, Freiburg im Breisgau, Germany; 6 Department of Surgical Medical Sciences and Advanced Technologies “G.F. Ingrassia” - Endovascular Neurosurgery, University of Catania, Catania, Italy; 7 Endovascular Neurosurgery, Pia Fondazione Cardinale Giovanni Panico Hospital, Tricase, LE Italy; 8 Department of Neurosurgery, University Medical Center Göttingen, Robert-Koch-Straße 40, 37075 Göttingen, Germany; 9 Department of Neurosurgery, King’s College Hospital NHS Foundation Trust and Department of Clinical Neurosciences, Institute of Psychiatry, King’s College London, London, UK; 10 N.N. Burdenko National Medical Research Center of Neurosurgery, Moscow, Russia; 11 Department of Neurosurgery, University of Helsinki and Helsinki University Hospital, Helsinki, Finland; 12 Neurosurgery Unit, Department of Neuroscience, ASST Grande Ospedale Metropolitano Niguarda, Milan, Italy; 13 Department of Neurosurgery, Inselspital, Bern University Hospital, University of Bern, Bern, Switzerland; 14 Department of Neurosurgery, Kepler University Hospital, Johannes Kepler University, Linz, Austria; 15 Department of Neurosurgery, Radboudumc Medical Center, Nijmegen, the Netherlands

**Keywords:** Assessment, Competency-based education, Entrustable professional activities, Patient care, Practice-based learning and improvement, Vascular neurosurgery

## Abstract

**Background:**

Entrustable professional activities (EPAs) represent an assessment framework with an increased focus on competency-based assessment. Originally developed and adopted for undergraduate medical education, concerns over resident ability to practice effectively after graduation have led to its implementation in residency training but yet not in vascular neurosurgery. Subjective assessment of resident or fellow performance can be problematic, and thus, we aim to define core EPAs for neurosurgical vascular training.

**Methods:**

We used a nominal group technique in a multistep interaction between a team of experienced neurovascular specialists and a medical educator to identify relevant EPAs. Panel members provided feedback on the EPAs until they reached consent.

**Results:**

The process produced seven core procedural EPAs for vascular residency and fellowship training, non-complex aneurysm surgery, complex aneurysm surgery, bypass surgery, arteriovenous malformation resection, spinal dural fistula surgery, perioperative management, and clinical decision-making.

**Conclusion:**

These seven EPAs for vascular neurosurgical training may support and guide the neurosurgical society in the development and implementation of EPAs as an evaluation tool and incorporate entrustment decisions in their training programs.

## Background

The treatment strategy for intracranial vascular pathologies has changed over the past 3 decades due to advances in endovascular and radiosurgical techniques. This transformation poses challenges to the future of microsurgery due to a significant decrease of surgical procedures. Experts nevertheless foresee a lasting role for open microsurgery in vascular neurosurgery [2, 4]. Microsurgery is of particular value in the treatment of arteriovenous malformations, complex aneurysms, and revascularization or flow replacement strategies, but there will be an equilibrium on non-ruptured aneurysms as well. This means a reduction in caseload and a shift towards more complex pathology.

Due to the growing complexity and centralization of care, a good microvascular neurosurgeon will have to function in a collaborative team of endovascular specialists and vascular neurologists in a tertiary care setting. These developments will require adaptation for vascular training since traditional training and examination methods of timed exposure and knowledge assessment cannot fully measure and guarantee the required competencies. Medical education has shifted to a competency-based system over the years, to educate and evaluate trainees more effectively and efficiently. Competencies are defined in so-called entrustable professional activities (EPAs). The Royal College of Physicians and Surgeons of Canada has defined two vascular EPAs: “Performing surgery for patients with an intracranial aneurysm” and “Performing surgery for patients with spontaneous intracerebral hemorrhage with or without an underlying vascular malformation” [5]. However, they do not include EPAs on complex vascular pathology, which presents a significant part of the surgical spectrum. The integration of the competency-based system into the traditional training would provide additional possibilities to ensure a high level of competency and skilled vascular neurosurgeons in the near future. Hence, the aim of this work is to develop a competency-based framework for general and complex vascular pathology to assist in resident and fellowship training.

## Methods

### Definitions

#### Entrustable professional activities

In order to operationalize a competency-based medical education program, curriculum development is based around EPAs. Where competencies describe the qualities of an individual person, EPAs describe the work that the professional must do [7]. EPAs require the professional to integrate multiple competencies from several domains that focus on individuals and their knowledge, skills, and attitudes.

#### Entrustment level scale

The entrustment level scale evaluates to what extend a trainee can be trusted to execute the EPA which is the focus of assessment. Although adjusted versions exist, a five-level entrustment scale is most commonly used [1, 7]:

1. Observation but no execution, even with direct supervision
2. Execution with direct, proactive supervision
3. Execution with reactive supervision, i.e., on request and quickly available
4. Supervision at a distance and/or post hoc
5. Supervision provided by the trainee to more junior colleagues

### Nominal group technique

We convened a group of cerebrovascular specialists in a six-stage approach to gain consensus on the EPAs [3]. (1) The first group consisted of two neurosurgeons (JHVL, HJS), one medical education specialist (BM), and a hybrid neurosurgeon (HB), who participated in a nominal group technique on the identification of the EPAs. (2) Each specialist was involved in direct patient care, worked with residents or fellows, and was an experienced medical educator. This was followed by a group discussion of each EPA and its competencies until agreement among the experts was reached. (3) Two authors (JHVL, HB) drafted the EPAs based on analysis of the collected data. (4) Subsequently, a cerebrovascular specialist (DH) independently reviewed the EPAs and competencies in direct communication with the corresponding author (JHVL). (5) A multidisciplinary international group of cerebrovascular specialists (panel members of the EANS vascular section) reviewed the EPAs and competencies. (6) The first group of specialists then refined and finalized the EPAs based on the feedback. The finalized EPAs were approved by all specialists.

## Results

### Phases 1 to 3

There were eight candidate activities that were reduced to five during the first round of discussion and eventually increased to six after revision in the final round resulted in the aneurysm surgery activity being split. We excluded the following EPAs: “professional development” because it does not entail a unit of work and therefore does not qualify as an EPA and “spinal arteriovenous malformations” due to the low incidence.

### Phases 4 and 5

The multidisciplinary international group of cerebrovascular specialists (panel members of the EANS vascular section) revised all six activities. Of these activities, none required major revisions to the description, but all six required minor changes. The reviewers suggested adding a separate EPA for spinal dural fistulas. The reviewers did not discard any activities.

### Phase 6 (Final review)

The first group of specialists reviewed the work of the reviewer in detail, made minor changes to all six activities, and added an EPA for spinal dural fistulas. The authors unanimously agreed to accept the seven activities as EPAs.

We have defined the following six EPAs:

- EPA 1: Non-complex aneurysm surgery
- EPA 2: Complex aneurysm surgery
- EPA 3: Bypass surgery
- EPA 4: Arteriovenous malformation resection
- EPA 5: Spinal dural fistula
- EPA 6: Perioperative management
- EPA 7: Clinical decision-making

**Fig. 1:**
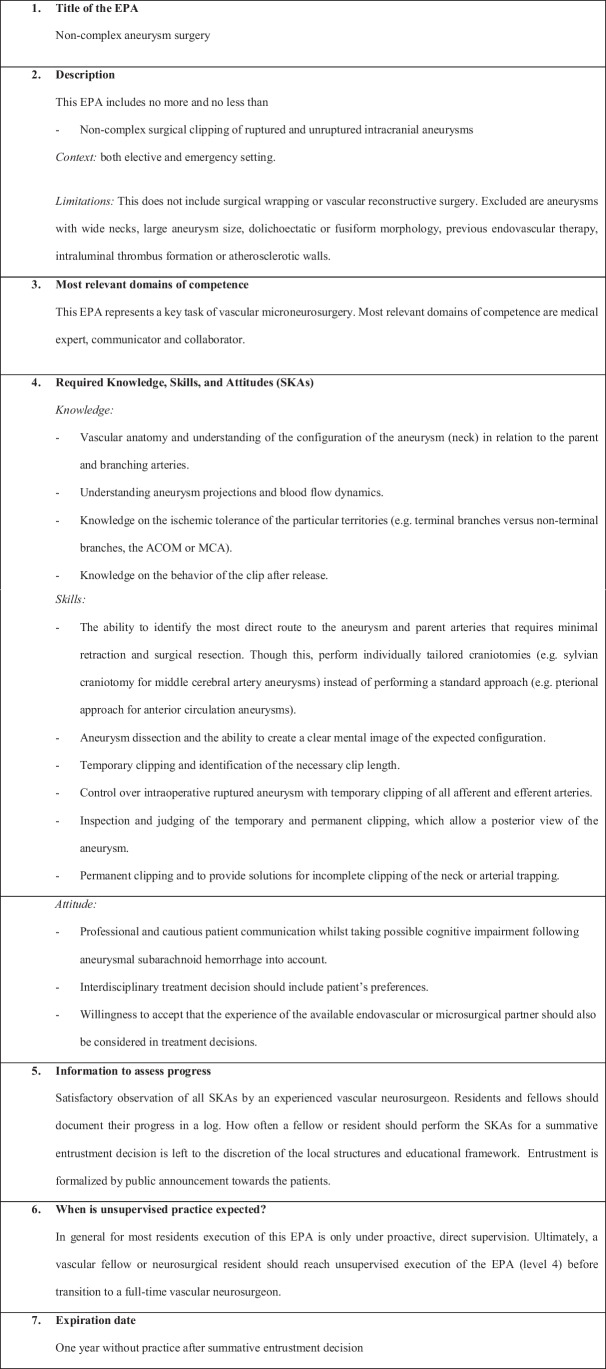
Non-complex aneurysm surgery

**Fig. 2:**
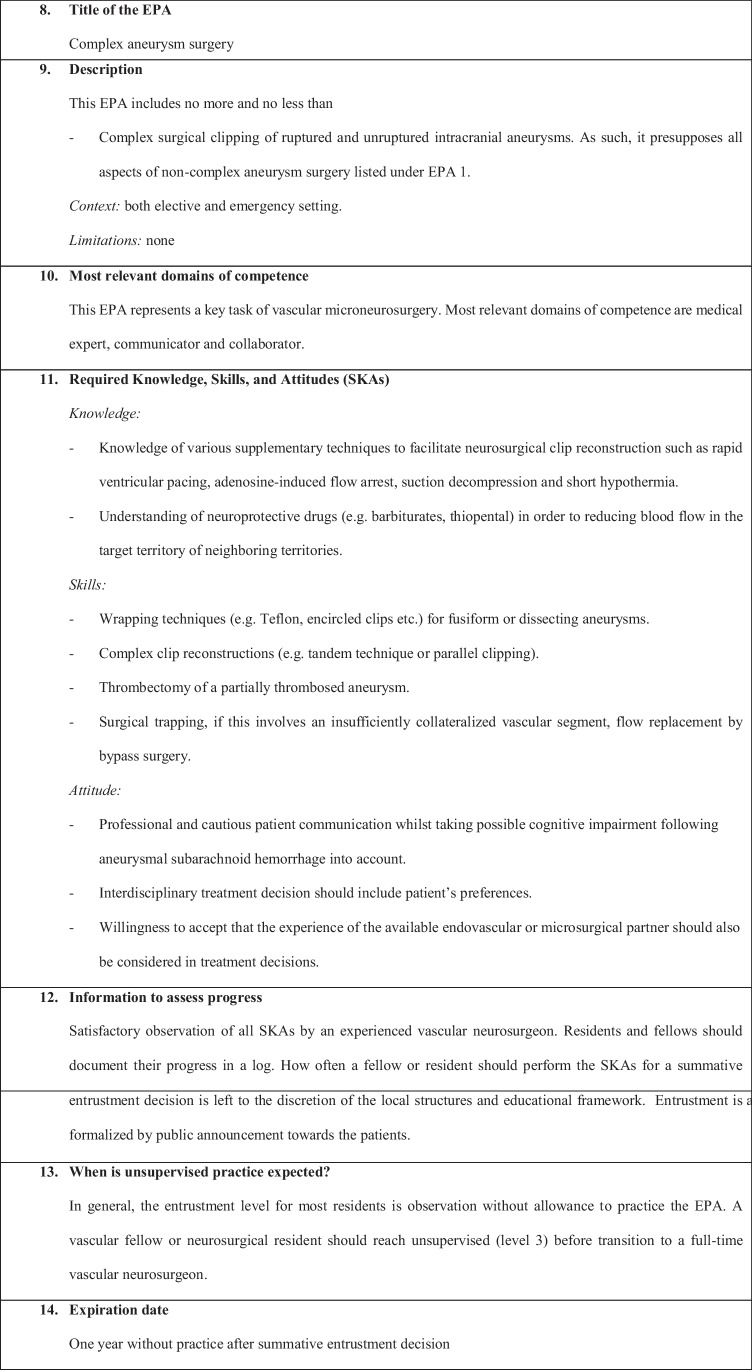
Complex aneurysm surgery

**Fig. 3:**
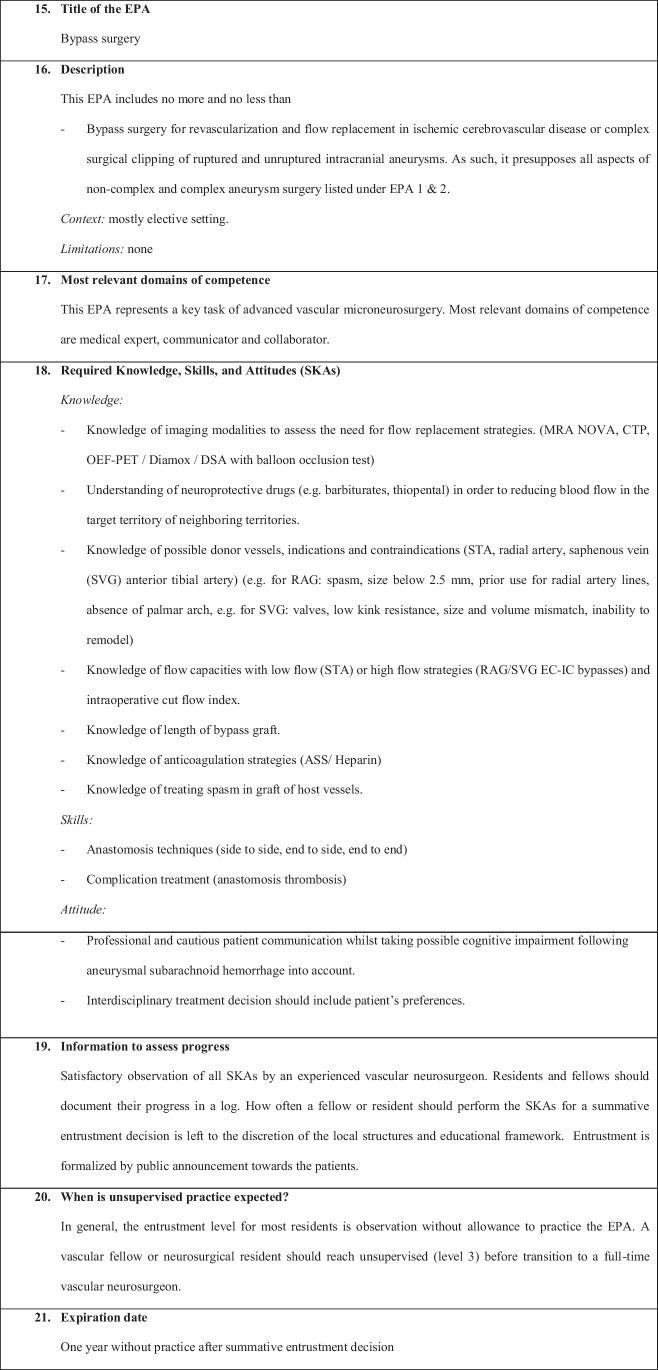
Bypass surgery

**Fig. 4:**
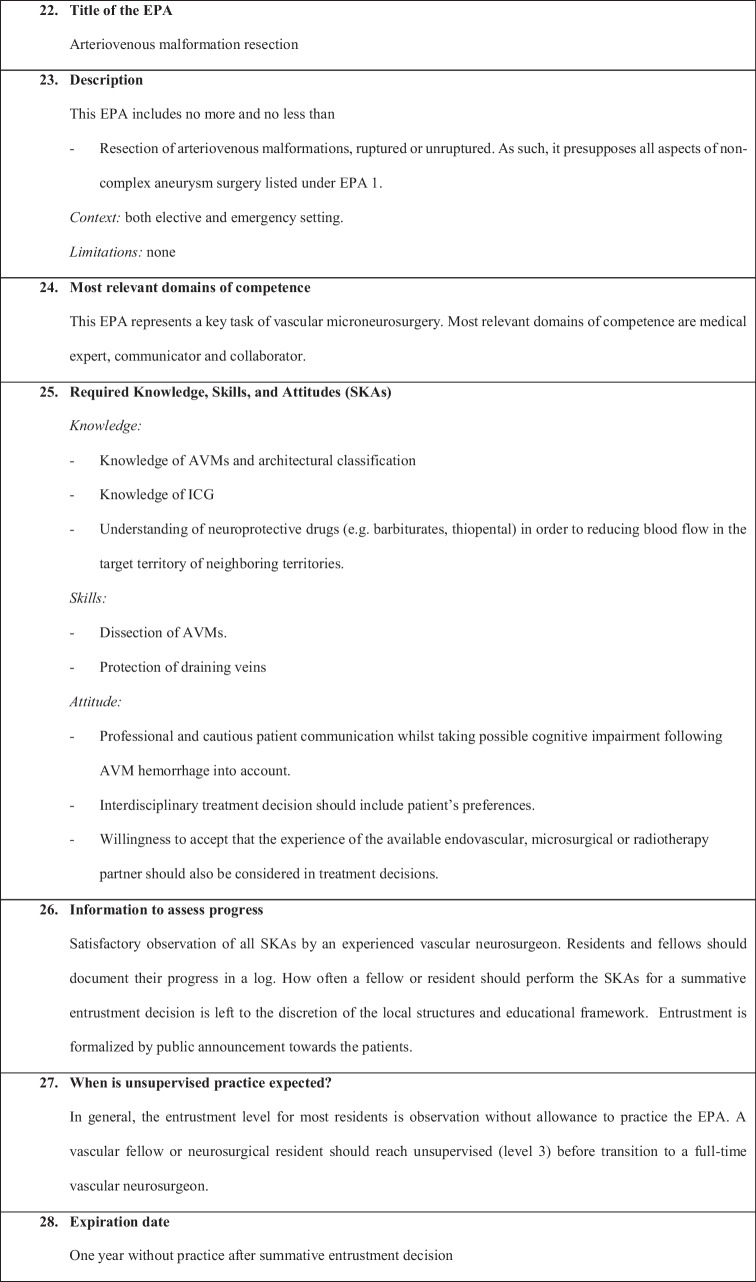
Arteriovenous malformation resection

**Fig. 5:**
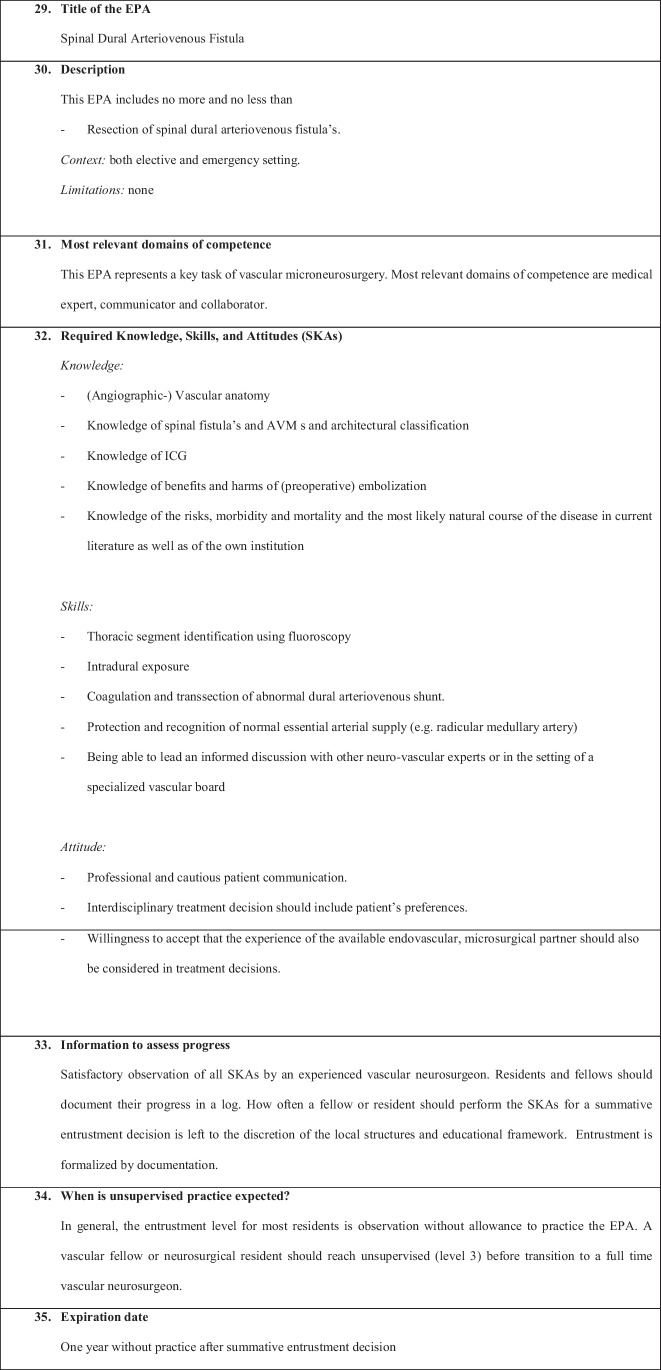
Spinal dural fistula

**Fig. 6:**
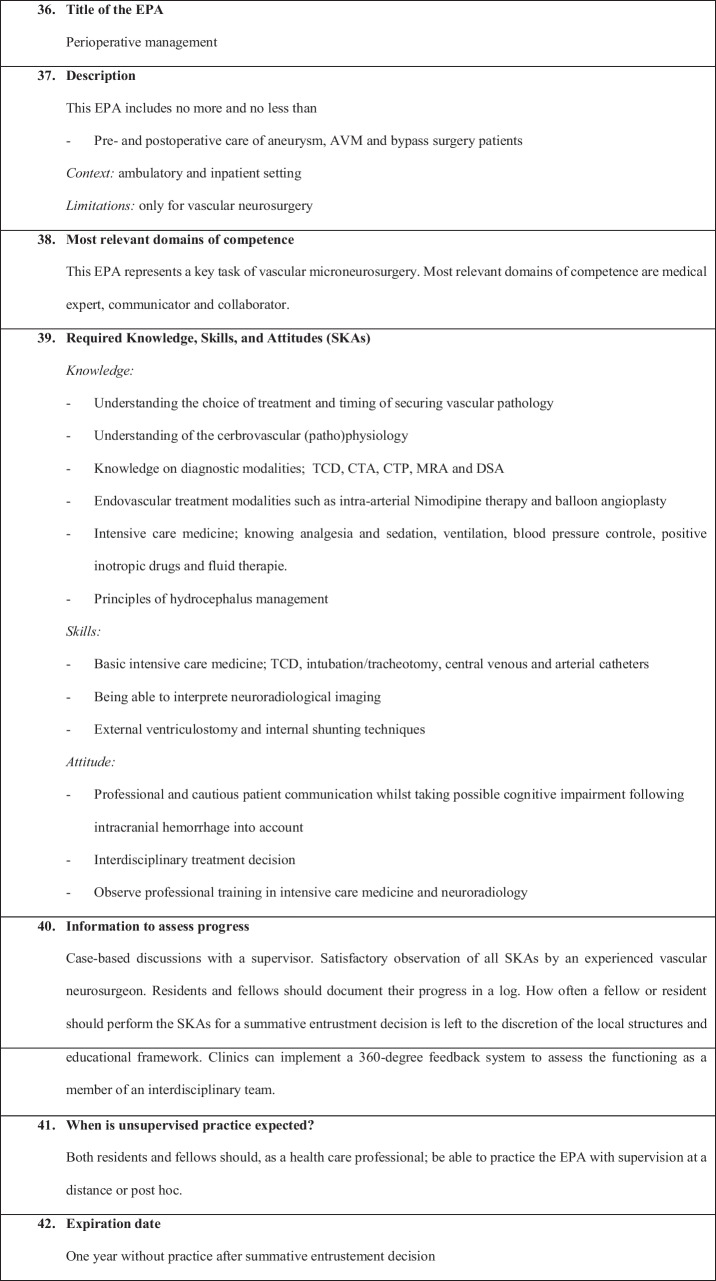
Perioperative management

**Fig. 7:**
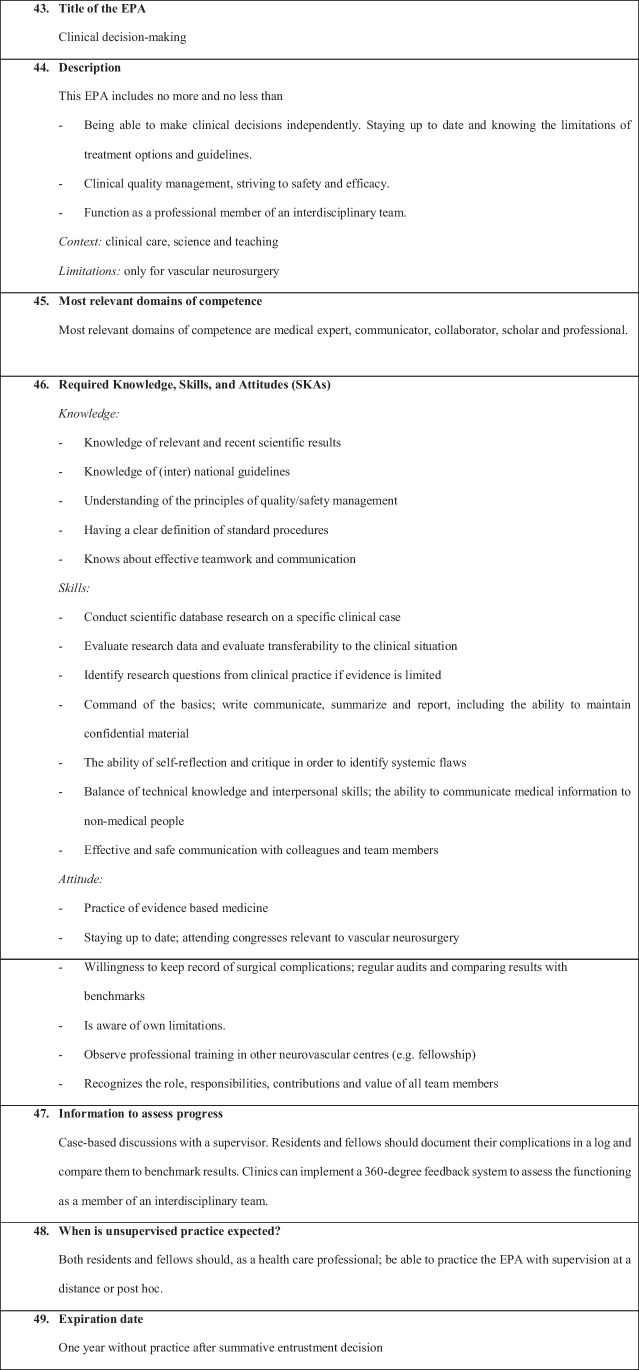
Clinical decision-making

Figures 1, 2, 3, 4, 5, 6, and 7 present a detailed description of each EPA.

## Discussion

We identified seven EPAs based upon their particular value in vascular microsurgery, for which we foresee a continuing role in vascular microsurgery. The present manuscript presents an attempt to add a competency-based system to traditional training in vascular neurosurgery.

Although this concept was initially developed to operationalize competency-based postgraduate medical education, it is now widely applied in health professions education [6, 8]. The Accreditation Council for Graduate Medical Education and the American Board of Surgery both have identified the need for competency-based skills assessments in surgical training, based upon a reported lack of confidence of surgical residents at the time of graduation [9]. By definition, EPAs allow for residents and fellows to achieve competence at different rates to increase surgical autonomy. This transition from traditional training and examination methods seems particular relevant for vascular microneurosurgery since technical advances in endovascular treatment modalities result in a reduction of caseload, whereas governments and patients increasingly demand metrics of competency for operative performance. EPAs present us with a possible framework for these metrics and can be added to residency and fellowship programs [9]. So far, there exists no threshold of competency metrics within the essential EPAs, but it is possible that governing bodies in the future will determine such a threshold for accredited residency and fellowship programs.

A recent study evaluated the appropriateness of the EPA “performing intracranial aneurysm surgery” for general residency and found that it was more suitable for a fellowship [8]. Since most of these activities are nowadays acquired during dedicated fellowships, these specialized EPAs are more suitable for this setting. The more general EPA’s numbers six and seven are also suitable for neurosurgery residency.

We are reasonably confident that this list of EPAs represents the core of vascular microsurgery. The EPAs proposed in the present article however are not meant to be infallible, and it is possible that other vascular specialists identify other relevant EPAs not included in this manuscript. The EPAs may also not fully reflect national or institutional priorities and may be adjusted to fit local vascular units.

These EPAs represent the core professional acts of vascular microsurgeons to provide safe and effective neurosurgical care. They thus are the activities that teachers should trust residents of vascular fellows to perform under varying degrees of supervision, and the supervision should ask whether he or she trusts the resident or fellow to perform these activities under what level of supervision. Medical educators should rate resident or fellow performance on EPAs using the before-mentioned entrustment scales. We do not aim to define training outcomes but rather focus on reaching an agreement on how to describe the stage a trainee is at for a given procedure. Continuing work would describe in more detail what a modern European cerebrovascular neurosurgeon should resemble in terms of experience. The neurosurgical society will need to determine the best methods of implementing the EPAs within vascular neurosurgery fellowship programs. [2] This study presents a foundation for the use and further development of specific EPAs for competency-based training of neurovascular microsurgical residents and fellows.

## Conclusion

These EPAs aid to focus on authentic professional work of vascular neurosurgeons and will represent the basis for assessment in vascular neurosurgery training.
